# Potential of the *Burkholderia cepacia* Complex to Produce 4-Hydroxy-3-Methyl-2-Alkyquinolines

**DOI:** 10.3389/fcimb.2019.00033

**Published:** 2019-02-28

**Authors:** Pauline M. L. Coulon, Marie-Christine Groleau, Eric Déziel

**Affiliations:** Institut Armand Frappier, Institut National de la Recherche Scientifique, Laval, QC, Canada

**Keywords:** *hmqABCDEFG* operon (*hmq*), quorum sensing, synteny, 4-hydroxy-2-alkylquinolines (HMAQ), alkylquinolones, *Pseudomonas aeruginosa*, *pqsABCDE* operon

## Abstract

A few *Burkholderia* species, especially *Burkholderia pseudomallei, Burkholderia thailandensis, Burkholderia ambifaria*, and *Burkholderia cepacia*, are known to produce and release various 4-hydroxy-3-methyl-2-alkylquinolines (HMAQs), a family of molecules analogous to the 4-hydroxy-2-alkylquinolines [aka 2-n-alkyl-4(1*H*)-quinolones] of *Pseudomonas aeruginosa*, which include the *Pseudomonas* quinolone signal (PQS). However, while these exoproducts play several roles in *P. aeruginosa* virulence and survival, the available literature is very limited on their distribution and function in *Burkholderia*. In this perspective article, we studied the distribution of the *hmqABCDEFG* operon, which encodes the enzymes involved in the biosynthesis of HMAQs, in the *Burkholderia cepacia* complex (Bcc) group. Based on the available sequence data, about one third of Bcc species carry a homolog of the *hmqABCDEFG*, and not all sequenced strains in a given species possess this operon. Looking at the synteny of genes surrounding the *hmqABCDEFG* operon, we found that for some species, the operon seems to have been deleted or replaced by other genes. Finally, we review the literature on the possible function of HMAQs. Understanding the Hmq system may provide clues concerning their functions in Bcc.

## Introduction

In 1992 several species originally classified as *Pseudomonas* defined the new *Burkholderia* genus (Yabuuchi et al., 1992). This genus now comprises more than 60 Gram-negative bacterial species, which fit into two clades: the plant-associated beneficial and environmental one or the pathogenic one (Surez-Moreno et al., 2012; Eberl and Vandamme, 2016). The plant-associated beneficial and environmental clade has recently been renamed *Paraburkholderia* because these bacteria lack biomolecular markers specific to pathogenic strains belonging to *Burkholderia* genus (Sawana et al., 2014). However, this split is controversial since some strains can be both pathogenic to animals and beneficial to plants (Eberl and Vandamme, 2016). The *Burkholderia* pathogenic clade is composed of plant, animal and human pathogens separated in two well-known groups: the “*pseudomallei*” group (*B. pseudomallei, B. mallei* and the environmental strain and study model *B. thailandensis*) and the opportunist pathogen species forming the *Burkholderia cepacia* complex (Bcc) (Surez-Moreno et al., 2012).

Bacteria belonging to the Bcc are mostly found in the rhizosphere, soil and water (Eberl and Vandamme, 2016; Loveridge et al., 2017). Bcc species are of interest in the industrial and agricultural fields, for instance for their potential in bioremediation (e.g., *Burkholderia vietnamiensis*), in plant growth promotion (e.g., *Burkholderia ambifaria*) and also for their capacity to produce an array of secondary metabolites [reviewed by (Vial et al., 2007)]. However, the realization that many Bcc species are responsible for serious chronic infections among immunosuppressed patients in general, notably those suffering from cystic fibrosis (CF) or chronic granulomatous disease (CGD), has put a hold on their biotechnological use, especially in agriculture (Vial et al., 2011). Indeed, because such infections are highly transmissible between patients and typically highly antibiotic resistant, they are often fatal (Gold et al., 1983; Gilligan, 1991; Govan et al., 1993; Speert et al., 1994; Govan and Deretic, 1996; LiPuma, 1998).

Bcc species produce various virulence determinants such as exopolysaccharides, siderophores and antimicrobials and can adopt several social behaviors such as swarming motility and biofilm formation. These are controlled by *quorum-sensing* (QS) (Kang et al., 1998; Lewenza et al., 1999; Richau et al., 2000; Huber et al., 2001; El-Banna and Winkelmann, 2002; Aguilar et al., 2003). QS is a cell-to-cell communication system used by bacterial populations to sense their density and thus control the transcription of certain genes in a coordinated manner (Fuqua and Greenberg, 1998). In fact, this signaling system allows bacteria to optimize colonization, to interact with their hosts and to better resist to stresses (Stewart and Costerton, 2001; Juhas et al., 2005).

**Figure 1:**
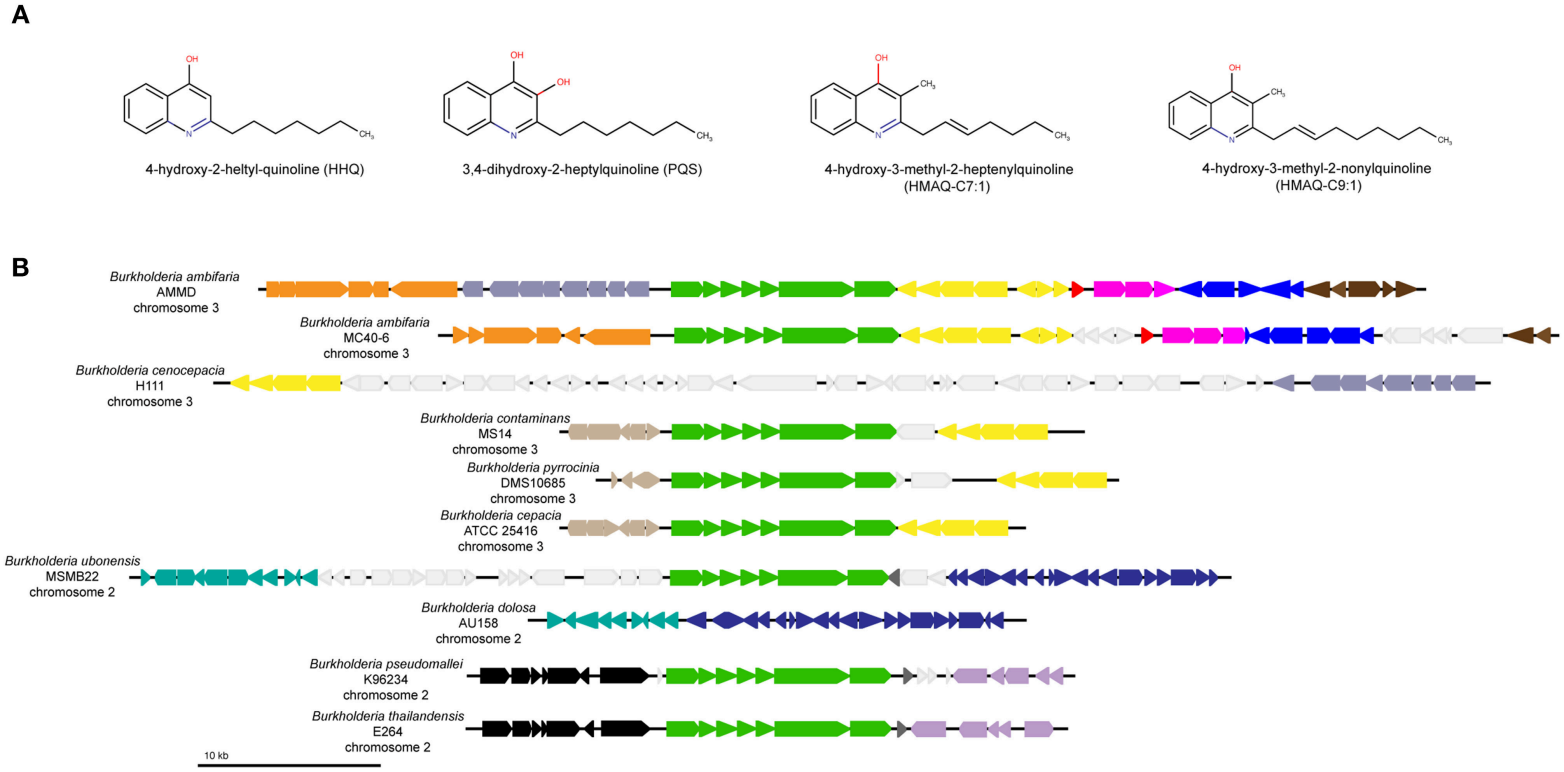
HAQ molecules and the synteny of genes surrounding the *hmqABCDEFG* operon in *Burkholderia cepacia* complex species. **(A)** Representation of HAQ molecules found in *P. aeruginosa* or/and in *Burkholderia*. **(B)** Synteny of *hmqABCDEFG* operon in Bcc: each color represents a different synteny group determined by MAUVE software. Only the closest genes of the *hmqABCDEFG* operon have been shown. The *hmqABCDEFG* operon is represented in green color. Light gray color represents variable metabolic genes without synteny.

In the Bcc, CepR is the main QS transcriptional regulator, which is activated by the autoinducing signal N-octanoyl-homoserine lactone (C_8_-HSL), the product of CepI (McKenney et al., 1995; Lewenza et al., 1999; Lewenza and Sokol, 2001). Depending on the species, Bcc have at least two *cep* (*lux-like*) systems (Choudhary et al., 2013), similar to the *las* and *rhl* systems in *Pseudomonas aeruginosa*. Interestingly, Pesci et al. (1999) reported a third signal they called the *Pseudomonas* quinolone signal (PQS) also produced in the latter bacterial species. It was then found that PQS actually belongs to a large family of extracellular molecules called 4-hydroxy-2-alkylquinolines (HAQ), also referred to as 2-n-alkyl-4(1*H*)-quinolones (Déziel et al., 2004). Gallagher et al. (2002) identified the *pqsABCDE* polycistronic operon as required for the production of these HAQs in *P. aeruginosa*. The *pqs* system was confirmed as a *bona fide* QS system when PQS, and its biosynthetic precursor 4-hydroxy-2-heptylquinoline (HHQ), were shown to act as autoinducing ligands of the transcriptional regulator MvfR (also known as PqsR) (Wade et al., 2005; Xiao et al., 2006). MvfR controls the expression of *pqsABCDE* (Déziel et al., 2004) resulting in an autoinducing loop. Unexpectedly, a few *Burkholderia* strains were then reported to produce minute levels of HHQ (but not PQS) and to carry a cluster of *pqsABCDE* homologs (Diggle et al., 2006). Subsequently, it was found that the main products in these *Burkholderia* strains are actually 4-hydroxy-3-methyl-2-alkylquinolines (HMAQs), principally distinct from *P. aeruginosa* HAQs by the presence of a methyl at the 3′ position and the predominance of an unsaturated alkyl side chain, among congeners (Vial et al., 2008), explaining why Diggle et al. (2006) had only detected very low concentrations of HHQ (Figure 1A). This biosynthetic operon was accordingly named *hmqABCDEFG*, with the additional encoded HmqF and HmqG, respectively, responsible for the unsaturation (Agarwal et al., 2012) and the methylation (Vial et al., 2008) of HMAQs. It should be mentioned that no congener corresponding to PQS, thus with a hydroxy substitution at the 3 position, has been detected in any *Burkholderia* culture, in agreement with the absence of a *pqsH* homolog. As with the *pqs* system in *P. aeruginosa*, interactions between the *hmq* and *cep* systems have been reported in *B. ambifaria* (Vial et al., 2008; Chapalain et al., 2017). While the production of some HMAQ congeners by a few *Burkholderia* isolates has been reported (Diggle et al., 2006; Mori et al., 2007; Vial et al., 2008; Kilani-Feki et al., 2011, 2012; Mahenthiralingam et al., 2011; Li et al., 2018), no studies have yet systematically investigated the prevalence of the *hmq* system and the capacity to produce HMAQs in the genus *Burkholderia*. In this perspective article, we review the knowledge on the *hmq* system, update the HMAQ biosynthesis pathway and investigate the distribution of the *hmqABCDEFG* operon in *Burkholderia* and especially in Bcc.

## Distribution of the *hmqABCDEFG* Operon in the *Burkholderia* Genus

Based on complete and draft genome sequences available in the *Burkholderia* Genome database (http://www.burkholderia.com), we have performed two complementary analyses with *hmqABCDEFG* of *B. ambifaria* strain AMMD as a reference: (1) an alignment sequence analysis () and (2) an orthology analysis. The BLAST analysis was run for the nucleic acid sequence of each gene composing the *hmq* operon, using the default settings (Johnson et al., 2008; Winsor et al., 2008). The orthology analysis consisted in examining the similarity of the protein sequence and the flanking gene regions by the pair-wise Diamond searches (Winsor et al., 2008; Buchfink et al., 2015). While that method may be more accurate than a BLAST analysis, an orthology analysis could miss some positive results if the *hmqABCDEFG* operon is in a different genomic location than the reference. Thus, a combination of both methods was selected.

Using the two analyses, we screened: (1) the seven genes composing the *hmqABCDEFG* operon based on the percentage of identity and (2) the species having all seven genes present in their genome. Sequences of the *hmqABCDEFG* operon are well conserved, with a nucleotide identity between 70 and 100%. Importantly, we only found instances of a *hmqG* homolog in a genome if it belonged to a *hmqABCDEFG* operon.

Based on both analysis methods, the *hmqABCDEFG* operon was found in 6 out of 21 Bcc species: *B. cepacia, B. ambifaria, B. pyrrocinia, B. ubonensis, B. contaminans*, and *B. stagnalis*, plus in some still unclassified *Burkholderia* spp.—having at least one sequenced genome present in the Burkholderia.com database (Table 1). Furthermore, some strains of *B. lata*, and *B. territorii* appear to have the *hmqABCDEFG* operon too, but this was only found via the BLAST analyses. Actually, the percentage of strains having the putative operon is higher by BLAST analysis than by orthology analysis. The *hmqABCDEFG* operon shows strong nucleic acid identity between the strains and within the genus *Burkholderia*. In fact, the average nucleic identities for each gene are between 78.5 and 83.9% compared to the *B. ambifaria* AMMD operon sequences (Supplementary Table 1). All these data show that (1) about one third of Bcc species carry the *hmqABCDEFG* operon and (2) within a species not all of the strains carry it.

**Table 1:** The *hmqABCDEFG* operon distribution in *Burkholderia cepacia* complex.

**Species**		**Bcc strains included in Burkholderia Genome DB [Complete genomes]**	**Analysis of the** ***hmqABCDEFG*** **operon's distribution by sequence**	
			**Orthology analysis (DIAMOND)**	**Alignment analysis (BLASTN)**
*Burkholderia cepacia* complex (Bcc)	*Burkholderia cepacia* (genomovar I)	337 [24]	42	77
	*Burkholderia multivorans* (genomovar II)	56 [7]	–	–
	*Burkholderia cenocepacia* (genomovar III)	243 [15]	–	–
	*Burkholderia stabilis* (genomovar IV)	–	–	–
	*Burkholderia vietnamiensis* (genomovar V)	41 [6]	–	–
	*Burkholderia dolosa* (genomovar VI)	2 [1]	–	–
	*Burkholderia ambifaria* (genomovar VII)	6 [2]	2	3
	*Burkholderia anthina* (genomovar VIII)	8 [–]	–	–
	*Burkholderia pyrrocinia* (genomovar IX)	4 [1]	1	3
	*Burkholderia ubonensis* (genomovar X)	292 [6]	75	283
	*Burkholderia latens* (BCC1)	2 [1]	–	–
	*Burkholderia diffusa* (BCC2)	12 [1]	–	–
	*Burkholderia arboris* (BCC3)	–	–	–
	*Burkholderia seminalis* (BCC7)	3 [1]	–	–
	*Burkholderia metallica* (BCC8)	1 [1]	–	–
	*Burkholderia lata* (group K)	4 [2]	–	2
	*Burkholderia contaminans* (group K, BCCAT)	7 [1]	2	3
	*Burkholderia pseudomultivorans*	9 [1]	–	–
	*Burkholderia stagnalis* (BCC B)	64 [1]	32	63
	*Burkholderia territorii* (BCC L)	33 [1]	–	2
	*Burkholderia paludis*	–		
	*Burkholderia* sp.	59 [18]	2	11
Total of Bcc strains		1257 [91]	166	447
*B. pseudomallei*		677 [75]	27	655
*B. thailandensis*		28 [15]	13	22
Total of *pseudomallei* group strains		705 [90]	284	677
Total of strains		1962 [181]	450	1123

*The distribution of the hmqABCDEFG operon have been determined by using the orthology analysis available on Burkholderia genome DB and BLAST from B. ambifaria AMMD nucleic acid sequence. Both methods have been used individual gene and only the strains having the all seven genes in their genome have been kept. For the BLAST analysis, only genes having a high identity with the reference have been kept. The sign “–” means that no sequences were available*.

A Bayesian phylogeny analysis, based on the *hmqABCDEFG* genes, shows extensive concordance with strain speciation, implying that this operon has been inherited from the common ancestor of *Burkholderia* rather than the results of recent gene transfer (Supplementary Figure 1). The presence of homologous operons in the evolutionary distant genera *Burkholderia* and *Pseudomonas* suggests a possible past horizontal gene transfer event, typically characterized (1) by a different %GC, (2) mobile elements insertion, and (3) genomic islands (Lawrence and Ochman, 2002; Juhas et al., 2009; Ravenhall et al., 2015). A similarly high %GC between *Burkholderia* (65%) and *P. aeruginosa* (67%) prevents the use of %GC to infer lateral gene transfer between these species. ISfinder (http://www-is.biotoul.fr) was used to find already known repeat sequences as indications of mobile elements insertion (Siguier et al., 2006). Islandviewer 4 (http://www.pathogenomics.sfu.ca/islandviewer) was used to predict genomic island on the third chromosome of *B. ambifaria* AMMD genome, and the second chromosome of *B. thailandensis* E264 and *B. pseudomallei* K96243 genomes (Bertelli et al., 2017). None of these methods revealed clear indications of horizontal gene transfer of the *hmqABCDEFG* operon and its surrounding genes.

## The Synteny of the *hmqABCDEFG* Operon in *Burkholderia*

Given that the *hmqABCDEFG* operon has a heterogeneous distribution in Bcc, we decided to examine its genomic context. The number of complete genome sequences being limited, the analyses was performed on *B. ambifaria* AMMD, *B. ambifaria* MC40-10, *B. cepacia* ATCC25416, *B. contaminans* MS14, *B. pyrrocinia* DSM 10685, *B. ubonensis* MSMB22, *B. pseudomallei* K96243, and *B. thailandensis* E264, all available in the *Burkholderia* Genome database (Winsor et al., 2008).

A multiple whole-genome alignment using progressiveMAUVE software was done to view conserved regions surrounding the *hmqABCDEFG* operon (Figure 1B) (Darling et al., 2010). By this analysis, we defined three groups: (1) *B. ambifaria*, (2) *B. contaminans, B. pyrrocinia, B. cepacia*, and (3) *B. ubonensis, B. cenocepacia* H111, and *B. dolosa* AU158 strains. Members of this third group lack the *hmqABCDEFG* operon in their genomes, are also included in the comparison to show a possible gene rearrangement. Synteny in genes surrounding the operon confirms the absence of the operon in *B. cenocepacia* H111 in which it is replaced by a number of metabolic genes. Also, a high similarity exists between the neighboring genes of *B. dolosa* AU158 and *B. ubonensis* MSMB22. In fact, in *B. dolosa* AU158 the *hmqABCDEFG* seems to have been deleted from or not yet integrated into the genome of this strain. The lack of available complete sequences limits the synteny analysis, more comparison could help us to have a better understanding of the *hmqABCDEFG* distribution.

## The Hmq Synthesis Pathway in *Burkholderia*

**Figure 2:**
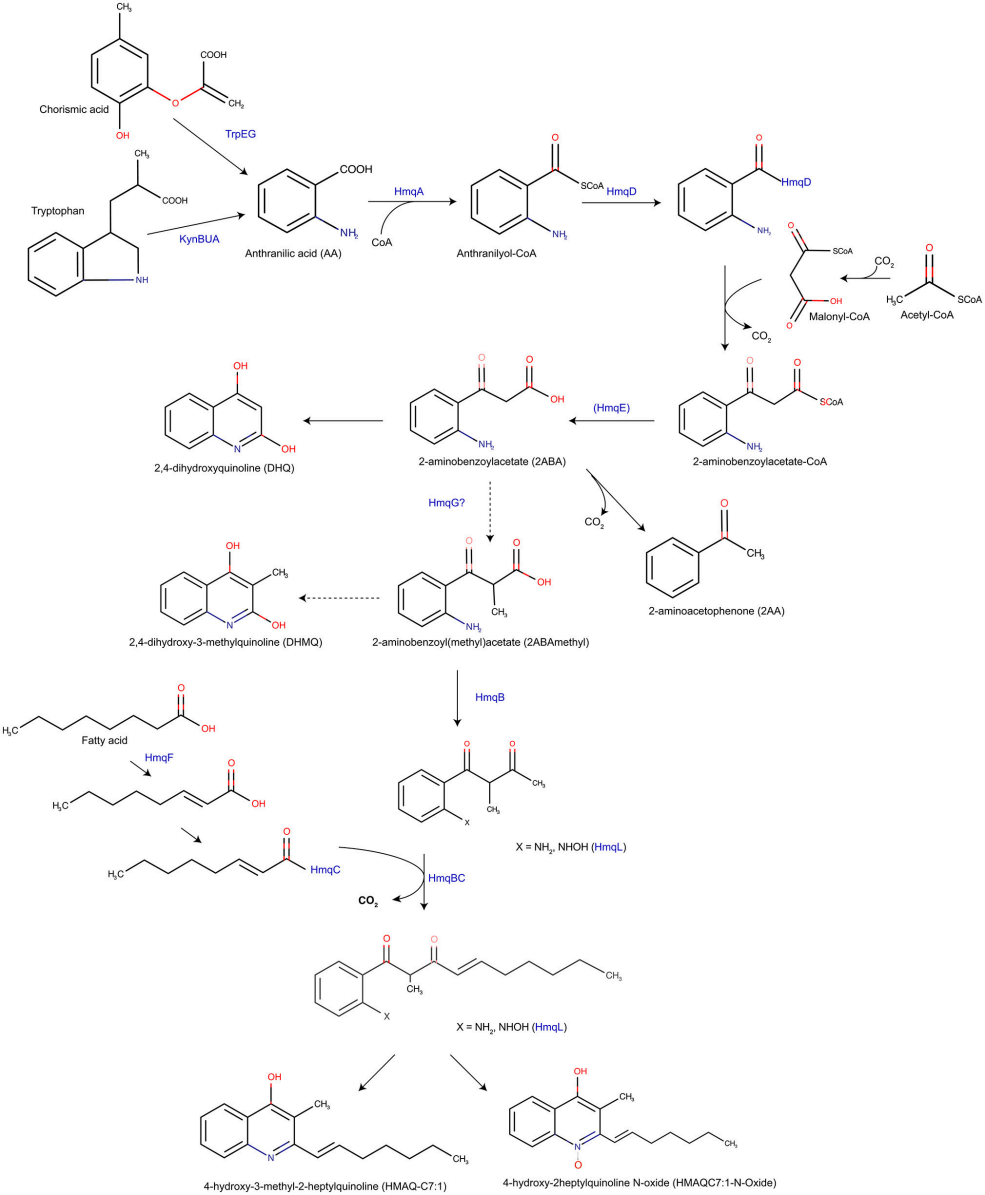
An updated version of the Hmq biosynthesis pathway based on Vial et al. (2008). Proposed Hmq biosynthesis pathway, based on HAQ biosynthesis in *P. aeruginosa*, produces HHQ and HMAQ both with a saturated or unsaturated acyl chain. HmqF adds the unsaturation on the fatty acid. HmqG may methylate 2-ABA to form 2-ABAmethyl which will form either HMAQ and HMAQ-N-oxides but also DHMQ in lower quantity. Note that only *B. thailandensis* is able to produce HQNO molecules.

In *Burkholderia*, the hmq system was found first on the second chromosome of four *Burkholderia pseudomallei* (K96243, 576, 10276, 844)*, B. thailandensis* E30 and on the third chromosome of *B. cenocepacia* J415, to be homologous to the *Pseudomonas* Quinoline Signal (PQS) system found in *P. aeruginosa* (Diggle et al., 2006). The hmq system produces 4-hydroxy-2-heptylquinoline (HHQ) but not 3,4-dihydroxy-2-heptylquinoline (PQS) signaling molecules (Diggle et al., 2006). In fact, the strains produce the methylated 4-hydroxy-3-methyl-2-alkylquinolines (HMAQs) instead of PQS molecules (Vial et al., 2008). The *hmqABCDEFG* operon encodes the *hmq* system and has two more genes than the *pqsABCDE* operon in *P. aeruginosa* meaning that the HMAQ biosynthesis and PQS biosynthesis pathways are similar except for the HmqF and HmqG additional functions [Figure 2; (Vial et al., 2008)].

In fact, the putative anthraniloyl-CoA synthase encoded by *hmqA*, allows anthraniloyl-CoA synthesis from anthranillic acid, coming from tryptophan or chorismic acid by the KynABU catabolism pathway and the TrpEG biosynthesis pathway, respectively (Farrow and Pesci, 2017). The putative 3-oxoacyl-ACP-synthase (HmqD), binding the anthraniloyl-CoA to transfer a SCoA fatty acid or an acetate to form 2-aminobenzoylacetate-CoA (Zhang et al., 2008; Dulcey et al., 2013). In addition, playing an active role in QS, the putative metallo-β-lactamase HmqE should favor the HHQ pathway production, because of the phenotype restoration in a *pqsE* mutant by adding *hmqE* (Diggle et al., 2006; Farrow et al., 2008; Rampioni et al., 2010, 2016; Folch et al., 2013; Drees and Fetzner, 2015). However, a *pqsE* mutant is still able to produce HHQ/PQS showing that PqsE is not been essential for PQS biosynthesis. Moreover, the β-ceto-decanoyl-ACP synthases HmqB and HmqC should form a complex like PqsBC [5DWZ; www.rcbs.org; Drees et al., 2016], considering the high similarity between the N-terminal amino acid sequence between HmqB and PqsB. By protein sequence analysis performed with Chimera [UCSF Chimera; (Pettersen et al., 2004)], this conserved amino acid sequence should allow the interaction between HmqB and HmqC by forming the same structure PqsB/PqsC, presumably. The two supplementary genes *hmqF* and *hmqG* encode, respectively, putative AMP-dependant synthase ligase and methylase. HmqF is responsible for the unsaturation on the alkyl sidechain while HmqG is responsible for the methylation of the molecule (Vial et al., 2008; Agarwal et al., 2012).

*Burkholderia* species do not carry PqsH homologous in their genomes, which explains the absence of the synthesis of 3-hydroxyl-HHQ molecules, such as PQS (Déziel et al., 2004). However, HmqL, an homolog of PqsL, has been found in *B. thailandensis* E264. This protein is a putative flavin adenine dinucleotide mono-oxygenase allowing the production of N-oxide HMAQs (Vial et al., 2008; Butt et al., 2016).

## HMAQs Production

The first HMAQ congener reported, named burkholone, was identified in culture broth of the environmental strain *Burkholderia* sp. QN15488 (Mori et al., 2007). Vial et al. (2008) described the production of three HMAQ families in *B. pseudomallei* 1026b, *B. thailandensis* E264, and *B. ambifaria* HSJ1, for a total of 19 different HMAQ congeners. This is without mentioning congeners without a methyl at the 3 position, thus similar to those produced by *P. aeruginosa* (Déziel et al., 2004). While it was originally believed that among Bcc species, only clinical *B. ambifaria* strains produce these molecules (Vial et al., 2008, 2009), it was later reported that environmental *B. ambifaria* AMMD and a few *B. cepacia* strains also produce HMAQs (Kilani-Feki et al., 2011, 2012; Mahenthiralingam et al., 2011). However, these latter studies required large concentrations of culture extracts, maybe explaining the absence of detection in the original studies. This suggests disparities in production levels between strains, possibly reflecting the lack of knowledge on the culture conditions directing the production of these secondary metabolites. Recently, Li et al. (2018) isolated new HMAQs from the culture broth of the environmental strain *Burkholderia* sp. MBAF1239. It is expected that more HMAQs congeners will be reported, with advances in metabolomic studies [e.g., see Okada et al. (2016)].

## Regulation of Hmq System and Function of HMAQs

Recently, a LysR-type transcriptional regulator named ScmR was found to positively influence the transcription of the *hmqABCDEFG* operon in *B. thailandensis* E264 (Mao et al., 2017). We found that ScmR has a potential 91.6% homologous gene on a sequence portion and ortholog in *B. ambifaria* AMMD strain (BAMB_RS03575). While HAQs such as HHQ and PQS are QS autoinducers in *P. aeruginosa*, no *hmqABCDEFG* operon induction by HMAQs has been seen in *B. ambifaria* HSJ1 and *B. thailandensis* E264 (Pesci et al., 1999; Vial et al., 2008; Chapalain et al., 2017). However, the *hmqABCDEFG* operon appears indirectly repressed by the *cep* system, while production of acyl-HSL is up in a HMAQ-null mutant (Chapalain et al., 2017).

When present on the genome of Bcc strains, the *hmqABCDEFG* operon is located on the third chromosome except in *B. ubonensis* in which it is similar to the “*pseudomallei*” group, where it is located on chromosome 2. Its location on the third chromosome, defined by Agnoli et al. (2011) as a virulence megaplasmid, suggests that the *hmqABCDEFG* operon is involved in virulence in some Bcc species. For now, the only identified function of HMAQs is as antimicrobials (Kilani-Feki et al., 2011; Mahenthiralingam et al., 2011). The non-methylated HQNO plays role in *B. thailandensis* E264 by inhibiting cytochrome *bc1* and pyrimidine biosynthesis like in *P. aeruginosa* (Wu and Seyedsayamdost, 2017). Also, HMAQ-C9:1, the main congener produced by *B. thailandensis*, seems to dissipate the proton motive force and also inhibits pyrimidine biosynthesis act synergistically with HQNO to inhibit bacterial growth (Wu and Seyedsayamdost, 2017). The question remains open for the Bcc; in our previous work, HQNO family congeners were absent from *B. ambifaria* cultures, and we proposed this was explained by the absence of a PqsL homolog, the enzyme required for the biosynthesis of this family (Lépine et al., 2004; Vial et al., 2008). All our analyses performed since then on HMAQs produced by a range of Bcc strains support this assertion: the Bcc does not produce HQNO family H(M)AQs (unpublished). Since HMAQs affect C_8_-HSL production, this impacts QS phenotypes such as the production of siderophores and proteolytic activity (Vial et al., 2008). Because of the analogy with the HAQs, HMAQs might act as signaling molecules, be required to control many virulence factors and even have an immuno-modulating activity as described for other HAQs (Heeb et al., 2011; Price et al., 2018). In fact, in *P. aeruginosa*, HHQ synchronizes the bacterial population by inducing the production of PQS (Déziel et al., 2004). Furthermore, PQS and HHQ act as immunomodulator interacting with the peripheral blood mononuclear cells and the dendritic cells (Hooi et al., 2004; Skindersoe et al., 2009; Kim et al., 2010a,b). Price et al. (2018) have shown that three *B. pseudomallei* strains isolated from CF patients overexpressed the *hmqABCDEFG* operon compared to *B. pseudomallei* K96243. Theses strains could use HMAQs as antimicrobials in niche protection or to modulate the host immune response during infection (Price et al., 2018). Apart from signaling properties, HMAQ might have pharmaceutical potential by acting as an antimicrobial and as a cytotoxic antibiotic against IGF-I dependant cells in cancer progression (Mori et al., 2007; Li et al., 2018).

## Conclusion and Future Directions

Taking all these elements into consideration, it is important to understand the distribution of the *hmqABCDEFG* operon and its evolution within the Bcc. The *hmqABCDEFG* operon's presence is distributed within some species in the Bcc but not all of the sequenced strains in a given species have the operon. More sequencing data will be required to reach broader conclusions on the *hmqABCDEFG* operon distribution trends. Moreover, because available data suggest that clinical strains produce more HMAQs than the environmental ones, it will be necessary to assess this characteristic in more Bcc species, and in more strains by species.

Variant strains of *B. ambifaria* HSJ1 produce much less HMAQ than the wild type (Vial et al., 2009) even if they have 99.8% nucleotide identity with *B. ambifaria* HSJ1 and *B. ambifaria* AMMD genomes, suggesting that a negative regulator could control the Hmq system. It will be interesting to know if the QS regulation of HMAQ production could be a conserved feature in Bcc, as previously reported in *B. ambifaria* HSJ1, by testing different species (Chapalain et al., 2017).

Our understanding of the prevalence, regulation and functions of HMAQs is limited. Apart from the antimicrobial activity, no function has been discovered yet. However, HMAQs may have similar roles to HAQs produced by *P. aeruginosa* based on their analogy (Vial et al., 2008; Price et al., 2018).

Future studies of the regulation of the *hmq* system and the role of HMAQs are necessary to know if they play a role in virulence. This knowledge should provide a better understanding of *Burkholderia* especially in pathogens *B. pseudomallei* and Bcc strains.

## Author Contributions

PC: conception and design of the article and acquisition, analysis and interpretation of data; M-CG: experiments on hypothetical biosynthesis pathway; PC, M-CG, and ED: revision; ED: resources and funding.

### Conflict of Interest Statement

The authors declare that the research was conducted in the absence of any commercial or financial relationships that could be construed as a potential conflict of interest.
