# Supplementary material for: Potential of the Burkholderia cepacia Complex to Produce 4-Hydroxy-3-Methyl-2-Alkyquinolines
Source: Front Cell Infect Microbiol. 2019 Feb 28;9:33. doi: 10.3389/fcimb.2019.00033 (PMC6403149; doi:10.3389/fcimb.2019.00033)
Supplement: Supplementary file 1 [file Data_Sheet_1.pdf]

Supplementary Material

Potential of the *Burkholderia cepacia* complex to produce 4-hydroxy-3-methyl-2-alkyquinolines

Pauline ML Coulon[1], Marie-Christine Groleau[1] and Eric Déziel[1*]

[1]INRS - Institut Armand Frappier, Laval, Québec, Canada

*** Correspondence:**
Eric Déziel
eric.deziel@iaf.inrs.ca

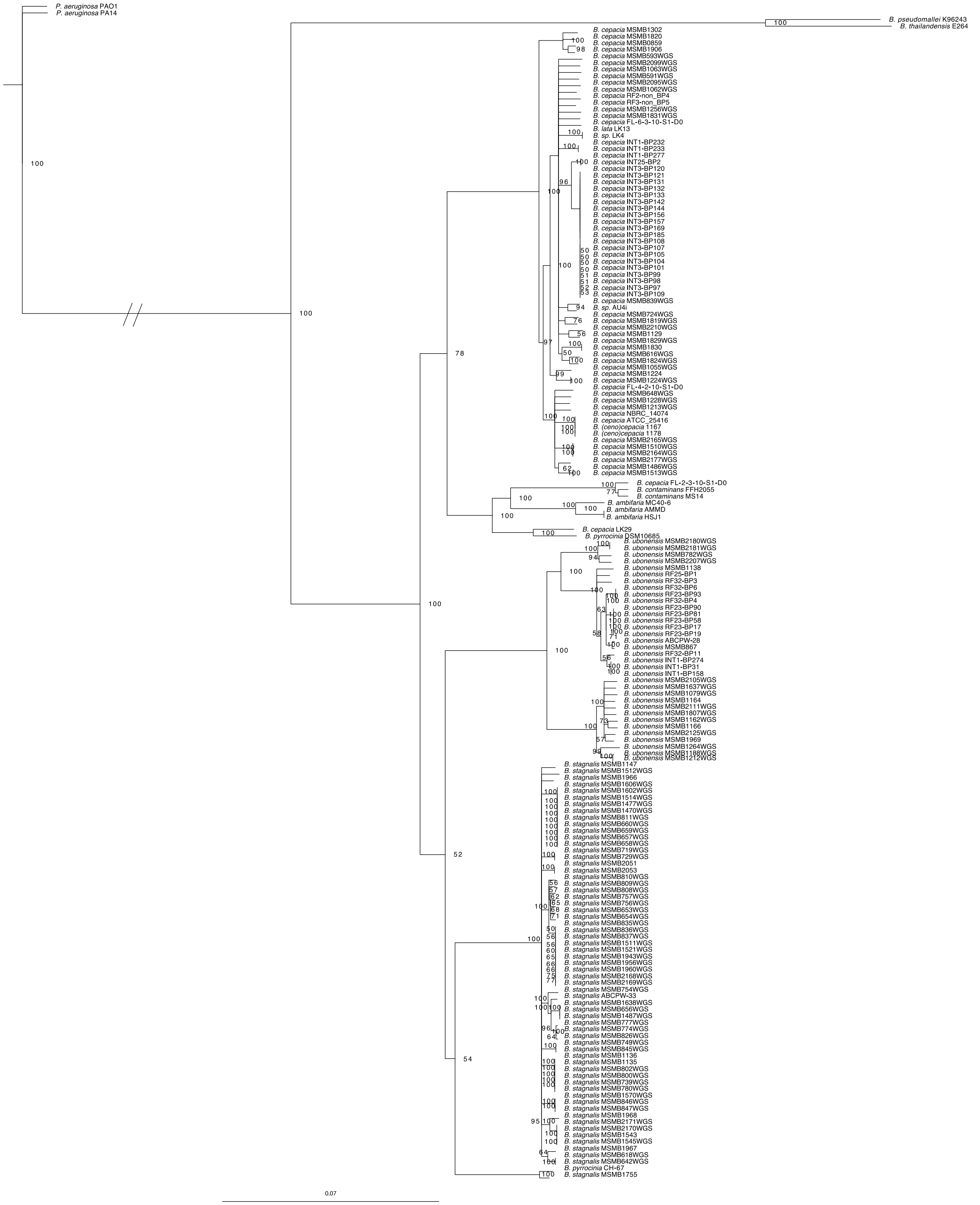

Supplementary Figure 1. Phylogenetic tree of strains of *Burkholderia* and the related species *P. aeruginosa* was generated in Mr Bayes based on the nucleic acids sequence of the complete *hmqABCDEFG* operon. A BLAST was run from *B. ambifaria* AMMD genome. The sequences were aligned by MUSCLE before analyzes by Mr.Bayes using the quick start settings on 9.000.000 generations. The p-value of the branches split was approximately 0.03.

Supplementary Table 1. Nucleotide identify of the *hmqABCDEFG* operon in Bcc, *B. pseudomallei* and *B. thailandensis*. Nucleotide identities are the average for each gene within the same species compared to the sequences of *B. ambifaria* AMMD. *The *hmqF* sequence was split (1-1754 bp and 1699-3819 bp) to be able to run the BLAST analysis on Burkholderia DB. These *hmqF* sequence fragments are overlapping, to be sure that both BLAST analyses have a part of the sequence in common.

Species		Number of genomes per species	Average nucleic identity (%) ± STDEV							
			hmqA **(1656 bp)**	***hmqB*** **(1116 bp)**	***hmqC*** **(888 bp)**	***hmqD*** **(999 bp)**	***hmqE*** **(897 bp)**	***hmqF**** **(3819 bp)**		***hmqG*** **(2139 bp)**
								**1-1754 bp**	**1699-3819 bp**	
Burkholderia cepacia complex (Bcc)	*Burkholderia cepacia* (genomovar I)	77	91.3 ± 0.4	89.9 ± 0.9	92.3 ± 0.4	92.0 ± 0.6	92.4 ± 0.3	90.6 ± 0.7	89.6 ± 0.7	91.5 ± 0.7
	Burkholderia multivorans (genomovar II)	-	-	-	-	-	-	-	-	-
	Burkholderia cenocepacia (genomovar III)	-	-	-	-	-	-	-	-	-
	Burkholderia stabilis (genomovar IV)	-	-	-	-	-	-	-	-	-
	Burkholderia vietnamiensis (genomovar V)	-	-	-	-	-	-	-	-	-
	Burkholderia dolosa (genomovar VI)	-	-	-	-	-	-	-	-	-
	Burkholderia ambifaria (genomovar VII)	3	99.5 ± 0.9	99.4 ± 1.0	99.3 ± 1.2	99.2 ± 1.4	99.7 ± 0.9	99.6 ± 0.7	99.5 ± 0.9	99.1 ± 1.5
	Burkholderia anthina (genomovar VIII)	-	-	-	-	-	-	-	-	-
	Burkholderia pyrrocinia (genomovar IX)	3	93.2 ± 0.6	93.1 ± 2.1	93.9 ± 1.2	93.8 ± 1.8	93.7 ± 0.9	91.3 ± 2.3	90.6 ± 2.8	92.4 ± 1.5
	Burkholderia ubonensis (genomovar X)	283	88 ± 0.2	90.2 ± 0.2	91.6 ± 0.3	93.8 ± 0.2	90.2 ± 0.3	89.5 ± 2.8	87.7 ± 2.6	90.2 ± 0.2
	Burkholderia latens (BCC1)	-	-	-	-	-	-	-	-	-
	Burkholderia diffusa (BCC2)	-	-	-	-	-	-	-	-	-
	Burkholderia arboris (BCC3)	-	-	-	-	-	-	-	-	-
	Burkholderia seminalis (BCC7)	-	-	-	-	-	-	-	-	-
	Burkholderia metallica (BCC8)	-	-	-	-	-	-	-	-	-
	Burkholderia lata (group K)	2	91.6 ± 1.0	90.5 ± 2.4	92.4 ± 0.1	93.3 ± 2.6	92.8 ± 0.7	92.8 ± 4.1	90.8 ± 1.8	92.5 ± 1.4
	Burkholderia contaminans (group K. BCCAT)	3	92.6 ± 0.2	91.6 ± 0.4	92.9 ± 0.3	94.7 ± 0.2	93.5 ± 0.4	95.6 ± 0.2	91.7 ± 0.1	93.7 ± 0.1
	Burkholderia pseudomultivorans	-	-	-	-	-	-	-	-	-
	Burkholderia stagnalis (BCC B)	63	92.1 ± 0.1	90.0 ± 0.2	92.9 ± 0.3	92.5 ± 0.1	92.4 ± 0.3	89.9 ± 0.2	89.0 ± 0.1	91.0 ± 0.2
	Burkholderia territorii (BCC L)	2	90.5 ± 0	88.6 ± 0	92.3 ± 0	91.3 ± 0	92.1 ± 0	89.9 ± 0	89.8 ± 0	88.4 ± 0
	Burkholderia paludis		-	-	-	-	-	-	-	-
	Burkholderia sp.	11	81.5 ± 8.8	82.4 ± 8.3	84.2 ± 7.2	87.2 ± 5.8	82.6 ± 8.7	81.4 ± 7.8	82.2 ± 6.4	81.6 ± 7.4
B. pseudomallei		655	77.4 ± 0.1	82.3 ± 0.4	80.7 ± 0.1	83.8 ± 0.1	77.1 ± 0.2	76.3 ± 0.1	77.9 ± 0.1	77.0 ± 0.1
B. thailandensis		22	77.9 ± 0.2	82.9 ± 0.5	80.8 ± 0.1	84.7 ± 0	76.7 ± 0.6	76.2 ± 0.1	77.9± 0.2	76.5 ± 0.2
